# Long-term HEV carriers without antibody seroconversion among eligible immunocompetent blood donors

**DOI:** 10.1038/s41426-018-0125-y

**Published:** 2018-07-05

**Authors:** Gui-Ping Wen, Chang-Rong Chen, Xiu-Yu Song, Zi-Min Tang, Wen-Fang Ji, Si-Ling Wang, Ke Zhang, Jun Zhang, Shan-Hai Ou, Zi-Zheng Zheng, Ning-Shao Xia

**Affiliations:** 1 State Key Laboratory of Molecular Vaccinology and Molecular Diagnostics, National Institute of Diagnostics and Vaccine Development in Infectious Diseases, School of Public Health, Xiamen University, Xiamen, Fujian 361102 PR China; 2 Xiamen Blood Service, Xiamen, Fujian 361005 PR China; 3 Xiamen Health Talent Service Center, Xiamen, Fujian 361003 China; 4 School of Life Sciences, Xiamen University, Xiamen, Fujian 361102 PR China

## Abstract

Hepatitis E virus (HEV) is emerging as a potential threat to the safety of blood transfusions. In many countries and regions endemic for HEV, such as China, blood donors are not routinely tested for HEV infection. In this study, 11747 eligible blood donors were screened for anti-HEV immunoglobulin M (IgM)/immunoglobulin G (IgG) and HEV RNA and antigen in China. Twenty-four donors who were positive for both HEV antigen and RNA were followed for ≥ 70 days, and none of these donors reported clinical hepatitis or illness. At least 1 follow-up sample was provided by 17 donors, including 10 with viremia and/or antigenemia for ≥ 70 days and 3 with antigen and RNA positivity for >90 days. Fourteen of the 17 donors did not present with an obvious serologic response during the follow-up period. These results differed from previous reports, in which viremia lasted for 68 days and elicited an antibody response. These donors showed atypical HEV infection progression that differed from that of hepatitis E patients. The presence of these donors presents a challenge for transfusion transmission screening.

## Introduction

Hepatitis E virus (HEV) represents an important global public health problem. HEV is a leading infectious cause of acute viral hepatitis in developing countries. Recently, hepatitis E (HE) has been recognized as an emerging and often undiagnosed disease in developed countries based on increasing reports of non-travel-associated, sporadic cases^1,2^.

HEV transmission usually occurs by eating and drinking contaminated food and water^3^. However, as evidenced by an increasing number of cases in Europe and Asia, HEV can also be transmitted via blood transfusion^4–7^. Prior studies have shown that the majority of HEV viremic blood donors are asymptomatic and seronegative for anti-HEV immunoglobulin M (IgM)/immunoglobulin G (IgG) at the time of donation^2,8–10^. Seroconversion was observed in follow-up samples from all donors, and viremia lasted for 68 days^2,8,9^. HEV infection occurred in 42–50% of recipients of HEV-contaminated blood products, and most of the donors of these blood products were asymptomatic at the time of donation^11,12^. A study of HEV in immigrants in Italy also suggested that asymptomatic HEV carriers play a potential role as human reservoirs, and the virus can be transmitted before the onset of the acute phase of HE^13^.

In China, studies of HEV in blood donors were mainly based on anti-HEV IgM marker and focused on the time point of donation^10,14^. In the present study, we conducted an investigation of HEV-related viremia and seropositivity among eligible blood donors by using HEV RNA, HEV antigen and anti-HEV IgM detection and analyzed the duration of HEV viremia in both HEV antigen- and HEV RNA-positive donors in Xiamen, a city in southeastern China.

## Results

### Characteristics of HEV RNA, HEV antigen, and anti-HEV antibodies among qualified blood donors

From December 2013 to February 2014, 5345 samples from eligible blood donors (donors with normal ALT levels and negative for HIV, HTLV, SYP, HBV, and HCV) were individually tested for HEV RNA, HEV antigen, anti-HEV IgM, and anti-HEV IgG. The overall prevalence rates of anti-HEV IgM and IgG were 0.71 and 22.96%, respectively. The prevalence rates of HEV RNA and HEV antigen were both 0.28% (15/5345). Eleven samples were positive for both HEV RNA and antigen. Four samples were only HEV RNA positive, and 4 samples were only HEV-antigen positive. All donors positive for HEV antigen and/or HEV RNA were completely asymptomatic, lacked physically detectable symptoms of infection, and were negative for anti-HEV IgM. Most of these donors (16/19, 84.2%) were also negative for anti-HEV IgG at the time of donation.

Because of the good concordance between the HEV RNA and antigen tests (Supplementary Table S1), HEV antigen was independently used in the second phase of this investigation, in which 6402 additional eligible plasma samples collected from March 2014 to May 2014 were screened to identify more HEV pathogen-positive blood donors. Antigen-positive samples were subsequently tested for the other examined HEV markers. Among 15 HEV antigen-positive samples, 13 samples were also positive for HEV RNA in the second phase.

**Fig. 1:**
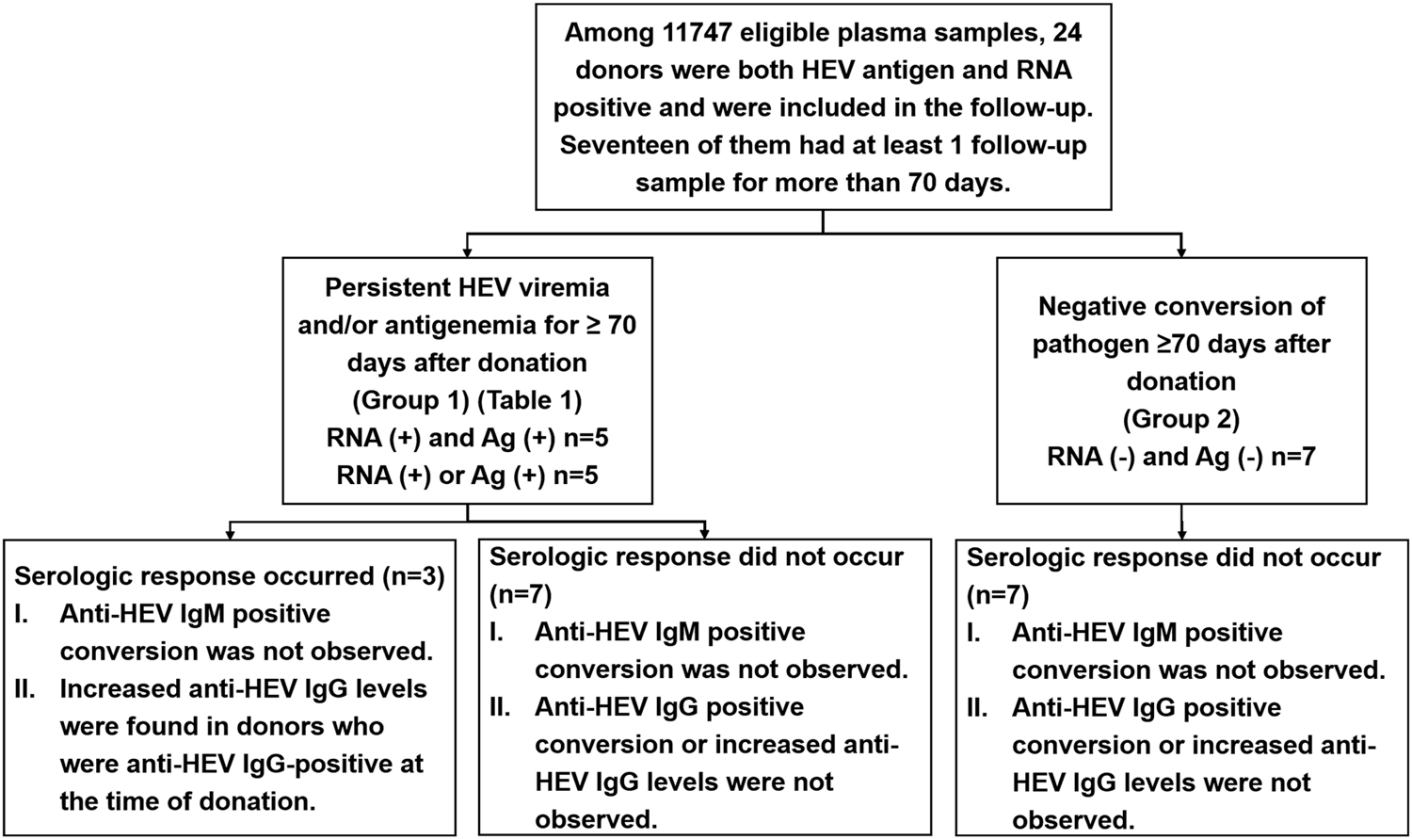
A schematic of the follow-up data from the 17 blood donors who were positive for both the HEV antigen and RNA (n=17). Among these 17 donors, 10 showed persistent viremia and/or antigenemia for ≥70 days after donation and increased anti-HEV IgG levels were observed in 3 donors. The other 7 donors showed negative conversion of pathogens (with both HEV antigen and RNA becoming negative) without a serologic response. Increasing IgG: increasing anti-HEV IgG level; Ag: HEV antigen

During the two phases of this investigation, from December 2013 to May 2014, 24 donors were identified as positive for both the HEV antigen and HEV RNA from among 11747 eligible blood donors (Fig. 1). In this study, similar to previously described HEV viremic donors, all 24 donors were negative for anti-HEV IgM, were asymptomatic, and lacked physically detectable symptoms of infection at the time of donation.

### Analyzing outcomes of HEV pathogen-positive blood donors

To analyze outcomes, all 24 donors were followed up for ≥ 70 days through telephone interviews every 2 weeks, and none of the donors reported clinical hepatitis or illness during the follow-up period. Seven donors declined to provide follow-up samples. At least 1 follow-up sample was provided by 17 donors, including 7 who exhibited clearance of the HEV RNA and antigen from their plasma 70 days post-donation. However, none of these donors presented with positive conversion of anti-HEV IgM/IgG or increasing anti-HEV IgG levels (Fig. 1, right path, Group 2; Supplementary Table S2).

The other 10 donors exhibited persistent viremia and/or antigenemia for more than 70 days after donation (Fig. 1, left path, Group 1; Table 1). The final pathogen-positive samples were collected at 71-185 (103 ± 35) days post-donation. At that time, 5 of these 10 donors (donors 1–5) were positive for both the HEV antigen and HEV RNA, and the remaining 5 donors (donors 6-10) were positive for a single pathogen marker (antigen or RNA) (Table 1). Donors 1, 2, and 3 were positive for both the antigen and RNA for more than 90 days (155, 92, and 91 days, respectively; Table 1). Only 1 of the 10 donors (donor 5) exhibited clearance of the HEV infection (with both the antigen and RNA becoming negative), which occurred on the 210^th^ day after donation (Table 1). At the follow-up time point, none of these 10 donors with persistent viremia and/or antigenemia exhibited positive conversion of anti-HEV IgM, which was also observed in the 7 donors with pathogen-negative conversion (Fig. 1, right path, Group 2; Supplementary Table S2). Three of these 10 donors (donors 3, 4, and 6) with a detectable plasma anti-HEV IgG level ( ≥ 0.077 WU/mL) at the time of donation presented with 4.12-fold, 1.56-fold, and 2.23-fold increases in the anti-HEV IgG levels, respectively, at the end of the follow-up period (Table 1). The remaining 7 donors maintained an anti-HEV IgG-negative status throughout the entire follow-up period, indicating that they did not undergo serological conversion during this time.

**Table 1:** Characteristics of the donors with long-term HEV viremia and/or antigenemia

Donors	Age^a^	Sex^b^	Career^c^	Sample	Days post-donation	RNA (copies/mL)^d^	HEV Ag (S/CO)^e^	Anti-HEV IgM (S/CO)^e^	Anti-HEV IgG (WU/mL)^f^
1	42	F	Other	1-1	1	5.55E + 05	1.192	0.023	0.039
				1-2	95	5.15E + 04	1.833	0.015	0.039
				1-3	155	5.05E + 04	1.958	0.046	0.039
2	35	F	Office clerk	2-1	1	1.41E + 05	12.275	0.15	0.039
				2-2	19	4.18E + 05	28.208	0.027	0.039
				2-3	92	2.05E + 05	23.192	0.023	0.039
3	38	F	Other	3-1	1	4.47E + 04	2.417	0.035	0.078
				3-2	91	2.32E + 04	1.45	0.004	0.321
4	47	F	Worker	4-1	1	2.18E + 05	1.087	0.127	0.404
				4-2	78	1.74E + 05	1.595	0.037	0.613
5	33	M	Doctor	5-1	1	7.22E + 04	1.075	0.004	0.039
				5-2	86	6.10E + 04	1.042	0.031	0.039
				5-3	210	neg	0.808	0.054	0.039
6	51	M	Civil servant	6-1	1	1.22E + 05	1.925	0.004	0.145
				6-2	101	neg	3.633	0.031	0.324
7	23	M	Other	7-1	1	7.15E + 05	4.592	0.015	0.039
				7-2	71	1.09E + 04	0.042	0.015	0.039
8	21	M	Worker	8-1	1	8.34E + 04	3.392	0.035	0.039
				8-2	69	1.69E + 04	0.9	0.05	0.039
				8-3	185	1.47E + 04	0.742	0.004	0.039
9	20	M	Worker	9-1	1	7.59E + 04	3.567	0.042	0.039
				9-2	91	neg	1.442	0.254	0.039
10	35	M	Office clerk	10-1	1	4.89E + 04	3.692	0.073	0.039
				10-2	81	2.74E + 04	0.125	0.031	0.039

^a^Age at time of donation

^b^Sex: F: female; M: male

^c^Potential careers included farmer, worker, office clerk, civil servant, student, teacher, doctor, other health worker, business staff, and other. Unemployed, self-employed, and housewife were included in the “other” category

^d^“neg” indicates negative for HEV RNA detection

^e^S/CO: signal to cut-off ratio. Values of S/CO ≥ 1 were identified as positive

^f^WU/mL: WHO units/mL. The levels of anti-HEV IgG in samples that were negative for anti-HEV IgG detection were set as 0.039 WU/mL

To analyze the immune systems of these donors, we measured the levels of IgM, IgG, and IgA in the plasma collected from the 10 donors with persistent HEV viremia and/or antigenemia. These 10 donors showed normal levels of antibodies in their plasma (Table 2). Common variable immunodeficiency diseases were not observed. All 10 donors were negative for HIV, HTLV, HCV, HDV, and HBV DNA and were positive for CMV-IgG. These 10 donors were in good health and did not receive immunosuppressive therapy or medicine. These results suggested that these donors were excluded from common immunocompromised patients. These donors were also negative for other pathogen markers, such as HAV, CA16, TB, CMV IgM, HV, and EV71 (data not shown). All 10 follow-up donors were deemed immunocompetent.

**Table 2:** IgM, IgG, and IgA quantification and other pathogen markers in donors with persistent HEV viremia

Donor	IgM (0.4–2.5 mg/ ml)	IgG (7-16 mg/ml)	IgA (0.8-4 mg/ml)	HIV RNA^a^	Anti-HIV (S/CO)^b^	Anti-HTLV (S/CO)	HAV-Ab^a^	HBV DNA^a^	HBsAg (S/CO)^b^	HBsAb (S/CO) ^b^	HBeAg (S/CO)^b^	HBeAb^a^	Anti-HCV (S/CO)^b^	HDV-IgG (S/CO)^b^	CMV-IgG (S/CO)^b^
1	1.61	13.27	2.58	−	0.11	0.01	+	−	0.22	33.98	0.02	−	0.17	0.05	16.79
2	2.05	14.14	2.69	−	0.14	0.03	+	−	0.08	32.97	4.71	+	0.03	0.05	13.16
3	1.98	15.81	3.03	−	0.05	0.02	+	−	0.76	33.60	0.04	−	0.03	0.05	21.34
4	1.90	13.65	3.07	−	0.48	0.02	+	−	0.29	17.75	0.03	−	0.18	0.04	11.17
5	1.17	13.91	2.90	−	0.04	0.03	+	−	0.06	0.13	0.03	−	0.03	0.05	3.62
6	0.54	14.86	3.02	−	0.07	0.03	+	−	0.08	2.39	0.04	−	0.04	0.08	12.44
7	1.31	13.38	2.60	−	0.07	0.03	+	−	0.10	0.18	0.04	+	0.03	0.04	3.86
8	1.05	13.51	2.75	−	0.06	0.03	−	−	0.18	2.62	0.02	−	0.03	0.04	8.10
9	1.23	14.86	2.72	−	0.03	0.01	+	−	0.27	33.98	0.04	−	0.03	0.05	16.79
10	1.24	14.11	2.76	−	0.03	0.01	−	−	0.55	20.04	0.01	−	0.03	0.04	14.51

^a^“−“ indicates negative for HIV RNA, HAV-Ab, HBV DNA, or HBeAb detection. “+“ indicates positive for HIV RNA, HAV-Ab, HBV DNA, or HBeAb detection

^b^S/CO: signal to cut-off ratio. Values of S/CO ≥ 1 were identified as positive

No donors had occupations that could potentially involve a high risk of exposure to an HEV-contaminated environment, such as farming or animal work. Moreover, no donors had known contact with a high-risk HEV-contaminated environment. All of them regularly consumed pork meat and shellfish.

There was no significant difference in HEV RNA or antigen levels between the 10 donors with long-term viremia/antigenemia and 7 donors who exhibited clearance of the virus. The HEV-related marker dynamics observed in the 10 donors with long-term viremia/antigenemia were similar to those observed in the 7 donors who exhibited clearance of the virus (Figure S1).

### Analysis of nucleotide sequences in the samples from persistent HEV viremia and/or antigenemia donors

**Fig. 2:**
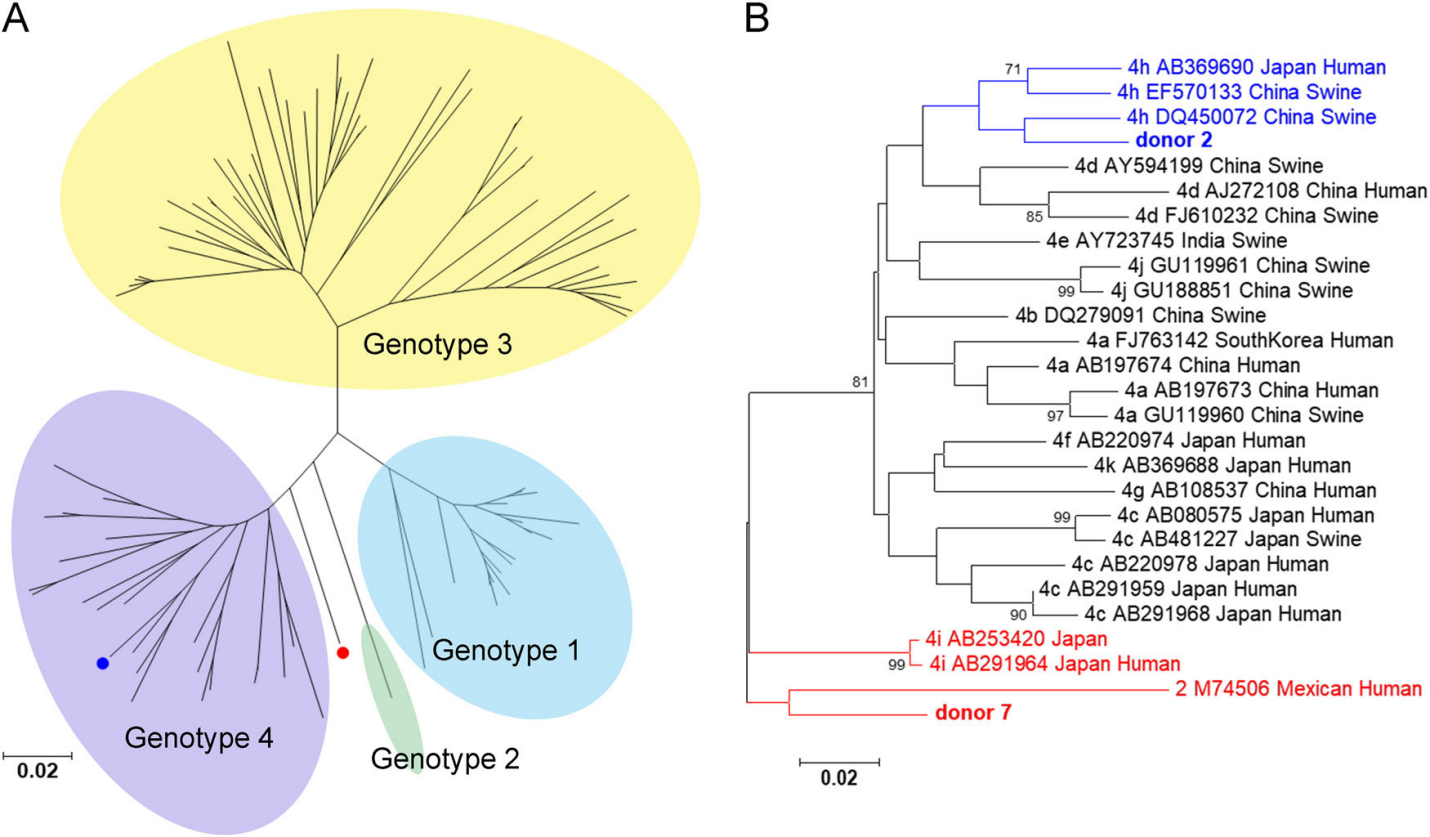
Phylogenetic tree analysis of the donors with long-term HEV viremia and/or antigenemia. Phylogenetic tree of genotypes 1–4 (**a**) and genotypes 2 and 4 alone (**b**). The HEV sequences were based on partial ORF2 nucleotide sequences from blood donors. The sequences from donors 2 and 7 are shown in blue and red, respectively. In the phylogenetic trees for genotypes 2 and 4 alone, the GenBank accession number, virus host, and country of detection are indicated for the reference sequences. The bootstrap values > 70 were shown

To analyze the viral sequences of donors with persistent HEV viremia and/or antigenemia, HEV RNA in the donor samples and follow-up samples was amplified with various primer pairs and methods as described in the Materials and methods section. The 150-nt ORF2 sequences from 2 donors (donors 2 and 7) were successfully amplified using a previously reported method^15,16^. In donor 2, the sequences isolated from the follow-up samples were the same as those isolated from the samples collected at the time of donation. In donor 7, the sequence was only successfully amplified in the sample collected at the time of donation and not in the follow-up sample. Phylogenetically, the sequence from donor 2 was closest to the 4 h HEV subtypes (GenBank accession number: DQ450072, Fig. 2, blue label). The sequence isolated from donor 7 was clustered between genotype 2 (GenBank accession number: M74506) and genotype 4i (GenBank accession number: AB291964 and AB253420) (Fig. 2, red label).

### Comparison of the progression of the HEV pathogen and the anti-HEV antibodies between donors with persistent HEV viremia and/or antigenemia and acute HE patients

To study the differences between donors with long-term HEV viremia and/or antigenemia and typical HE patients, we further compared the progression of HEV RNA, HEV antigen, anti-HEV IgM, and anti-HEV IgG between these two populations. The series samples from 20 typical acute HE patients were measured using the same methods applied to evaluate the blood donor samples.

**Fig. 3:**
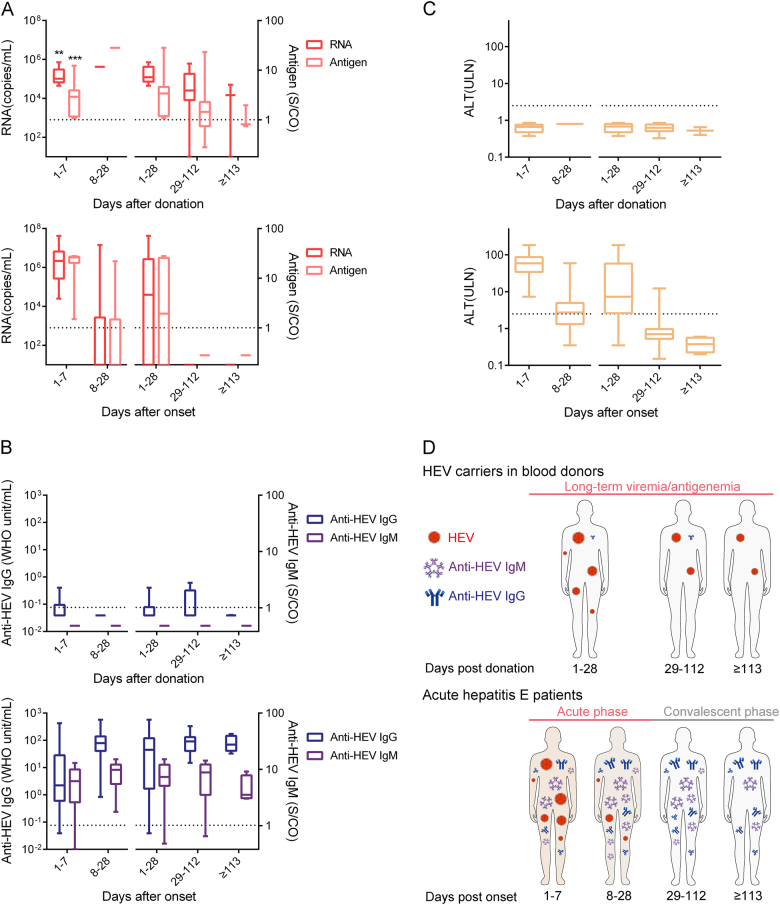
Dynamics of the HEV pathogen (RNA and antigen) (**a**), anti-HEV antibodies (IgM and IgG) (**b**) and ALT levels (**c**), and models (**d**) among 10 donors with long-term HEV viremia and/or antigenemia (upper panel) and 20 acute hepatitis E (HE) patients (lower panel) during the follow-up period. Samples were collected from 10 donors who presented with long-term HEV viremia and/or antigenemia from 1 to 7 days (*n* = 10), 8 to 28 days (*n* = 1), 1 to 28 days (n = 11), 29 to 112 days (*n* = 10), and ≥ 113 days (*n* = 3) after donation. Samples were also collected from 20 HE patients from 1 to 7 days (*n* = 17), 8 to 28 days (*n* = 24), 1 to 28 days (*n* = 41), 29 to 112 days (*n* = 28), and ≥ 113 days (*n* = 4) after the onset of symptoms. Dotted lines represent the cut-off levels for the HEV antigen and anti-HEV IgM/IgG values. Viral RNA, HEV antigen, anti-HEV IgM, and anti-HEV IgG values and ALT levels are presented as ranges (whiskers), interquartile ranges (boxes), and medians (lines within the boxes). The differences in RNA and antigen levels between blood donors from 1 to 7 days after donation and HE patients from 1 to 7 days after onset were analyzed using the Mann–Whitney *U* test. “*”*p* < 0.05; “**”*p* < 0.01; “***”*p* < 0.001

As shown in Fig. 3, donors with long-term HEV viremia and/or antigenemia showed atypical HEV infection progression that differed from that of HE patients. An undefined mode of HEV infection distinct from that of typical acute HE patients was observed among blood donors (Fig. 3d). In donors with long-term HEV viremia and/or antigenemia, HEV RNA and HEV antigen remained positive for up to 112 days after donation (Fig. 3a, upper panel). In acute HE patients, positivity for HEV RNA and HEV antigen lasted for ≤ 28 days with abnormal ALT levels, and virus clearance and ALT level normalization were achieved within 28 days after the onset of symptoms in all patients (Fig. 3a, c, lower panel). In other words, the viremic period was significantly longer for donors than for acute HE patients (*p* < 0.0001). Additionally, the levels of HEV RNA and antigen in the donors with long-term HEV viremia and/or antigenemia from 1 to 7 days after donation were significantly lower than those in acute HE patients from 1 to 7 days after HE onset (*p* < 0.005, Fig. 3a). Anti-HEV IgM was not detected in any donor (Fig. 3b, upper panel), but positivity for anti-HEV IgM and anti-HEV IgG was observed at the time of the first sample collection and persisted for 224 days after the onset of symptoms in most HE patients (Fig. 3b, lower panel). The anti-HEV IgG levels in donors were also significantly lower than those in patients who had recovered from acute HE (*p* < 0.0001).

## Discussion

Seroprevalence studies conducted in many countries have shown that the prevalence of HEV RNA in blood donors ranges from 0.012% to 0.6%^11,17,18^. Cases of transfusion-transmitted HEV infection have been reported in Asia and Europe^19^. Patients who require transfusion are often immunosuppressed or in poor health. In the UK, 75% of blood and blood components are administered to immunocompromised patients^19^. Blood donations contaminated with the HEV pathogen represent an obstacle for the administration of safe transfusions. The present study showed that the positive rate of HEV RNA was 0.28% (15/5345), which was higher than that previously reported among Chinese blood donors (0.04%)^10^, in which anti-HEV IgM was used as the major detection marker. Furthermore, the positive rate of HEV RNA shown in our study was also higher than most reported values from European countries^11,17,18^, which was consistent with the higher prevalence in China than in Europe.

In this study, we found that HEV infection progression in 10 pathogen-positive donors was inconsistent with that reported in previous studies and differed from that in typical HE patients. Among 11747 eligible blood donors, 24 donors were identified as positive for both HEV antigen and HEV RNA, with anti-HEV IgM negativity and normal ALT levels. These 24 donors were followed up for ≥ 70 days; none of them reported clinical hepatitis or illness, and 17 provided at least 1 follow-up sample ≥ 70 days after donation (Fig. 1). Ten of these 17 donors had HEV viremia and/or antigenemia ≥ 70 days after donation, and 3 were positive for both HEV antigen and RNA for more than 90 days (Fig. 1; Table 1). None of these donors had been exposed to or had contact with a high-risk HEV-contaminated environment, and all of them regularly consumed pork meat and shellfish. The sequences isolated from the follow-up samples of donor 2 were the same as those from the sample collected at the time of donation. Therefore, these donors may have long-term viremia and/or antigenemia. The period of viremia in these 10 donors was longer than that observed in previous reports, in which the duration of HEV viremia in blood donors was reported to be 68 days^2,8,9^. The duration of HEV viremia was considered to be 68 days and 46 days in Dutch and England blood donors, respectively^8,9^. A previous study showed that HEV viremia lasted a maximum of 52 days in blood donors in Germany^2^. Our study showed that HEV viremia could last up to 90 days in blood donors. Single HEV RNA was used to identify HEV-positive cases in these previous reports^2,8,9^, but both HEV antigen and RNA were used to identify HEV-positive cases in this study, further confirming that the viremic period could be ≥ 68 days in blood donors. These results suggest that the period of viremia in blood donors was longer than 28 days, as observed in acute HE patients with ALT elevations.

Anti-HEV IgM/IgG-positive conversions were not observed in the 17 donors who provided a sample during the follow-up period. Increased IgG levels were found in 3 donors who were positive for anti-HEV IgG at the time of donation. In acute HE patients, anti-HEV IgM positivity and anti-HEV IgG positivity were generally present with the onset of symptoms and persisted for approximately 224 days (Fig. 3). Previous reports have also demonstrated that anti-HEV IgM and anti-HEV IgG can persist in the serum for an average of 5 months and 24 months, respectively^20,21^. As a result, most of the follow-up samples were collected ≥ 70 days after donation in this study, and anti-HEV IgM seroconversion should have been observed at least in certain cases at that time. Anti-HEV IgM seroconversion did not occur in patients with reinfection, although anti-HEV IgG seroconversion or an increase in anti-HEV IgG levels should have been observed. This finding suggests that 14 of the 17 donors did not present with an obvious serologic response during the follow-up process. This phenomenon is inconsistent with findings described in previous reports, in which seroconversion occurred during the follow-up period of viremic donors who were seronegative at the time of donation^2,8,9^. Generally, HEV infection causes typical acute HE, but chronic HE was also found in immunosuppressed persons^22,23^. In these chronic HEV infection cases, HEV RNA and HEV antigen can persist for ≥ 6 months with abnormal ALT levels, and anti-HEV IgM/IgG positivity can be observed in most chronic HEV cases^22,23^. The HEV infection progression shown in this research with persistent HEV viremia and/or antigenemia and rare antibody responses differed from that observed in chronic HEV cases. Similar cases were also observed in a recent German study through retrospective analysis, and this study suggested that the blood products from these carriers lead to transfusion-transmitted HEV infection^24^.

Final infection clearance was not observed in the 10 donors with long-term viremia/antigenemia. An obvious serologic response was not observed in donors with pathogen-negative conversion. It is hypothesized that these 17 donors showed the same HEV infection pattern and that the 7 donors who showed infection clearance were in the late phase of this infection pattern.

In this study, we analyzed all 5345 samples in the first phase for HEV RNA. There was no significant difference in the HEV RNA levels between the single HEV RNA-positive and both HEV RNA- and HEV antigen-positive donors (Supplementary Figure S2). Differences in the HEV RNA levels were found between acute HE patients and HEV carriers in blood donors (Fig. 3). No patients or carriers were observed who were HEV antigen negative with HEV RNA levels of > 10^6^ copies/mL. All the cases with RNA level > 10^6^ copies/mL were found in acute HE patients who were also HEV antigen positive. Furthermore, all of these HE patients showed obvious antibody responses and virus clearance during the follow-up period. We speculated that the levels of HEV RNA and HEV antigen may be related to the induction of immune responses and virus clearance. Meanwhile, 7 donors who exhibited clearance of the virus did not report clinical symptoms and did not display serologic responses in the present study. Therefore, there may be an unknown mechanism of viral clearance in these HEV carriers different from that in acute HE patients.

In summary, this study identified immunocompetent HEV pathogen-positive donors with long-term viremia and/or antigenemia and without a serologic response or symptoms of hepatitis. None of these donors had been exposed to or had contact with a high-risk HEV-contaminated environment. These donors thus present a challenge for the screening of transfusion-transmitted infections.

## Materials and methods

### Sample collection

Plasma samples were collected from volunteers at the Xiamen Blood Station in China and tested for all routinely screened donor markers. The donors included in the study were negative for hepatitis B virus (HBV), hepatitis C virus (HCV), syphilis (SYP), and human immunodeficiency virus (HIV) and had normal alanine aminotransferase (ALT) levels without signs of hepatitis during routine donor screening in the blood station. From December 2013 to February 2014, 5345 eligible plasma samples were individually tested for all 4 HEV-related markers, including anti-HEV antibodies (IgM and IgG) and the HEV pathogen (RNA and antigen). Because of the good concordance found between the HEV RNA and antigen tests in this study, 6402 additional eligible plasma samples were tested for the HEV antigen. Only HEV antigen-positive samples were subsequently tested for HEV RNA, anti-HEV IgM, and anti-HEV IgG to identify additional HEV pathogen-positive donors from March to May 2014. All blood donors who were both HEV RNA and antigen positive were followed up for ≥ 70 days. The follow-up samples were also tested for all 4 HEV-related markers.

Samples from 20 acute HE patients with at least 1 follow-up serum sample were collected in Dongtai (Jiangsu Province, China) from December 2013 to May 2014. Acute HE cases were defined as both HEV RNA- and antigen-positive acute hepatitis patients with at least a 2.5-fold upper limit of normal (ULN) elevation in ALT levels who had suffered from a loss of appetite and/or fatigue for ≥ 3 days.

This study was designed and performed in accordance with the ethical guidelines of the 1975 Declaration of Helsinki. Written informed consent was obtained from all participants. This study was approved by the Ethics Committee of the Jiangsu Provincial Center for Disease Control and Prevention and the Xiamen Blood Service.

### HEV RNA detection using quantitative reverse transcript (RT) PCR

HEV RNA detection was performed using a real-time RT-PCR assay using primers that targeted nt 5302-5371 based on GenBank accession no. M73218 as previously reported^25–27^. Briefly, HEV RNA from individual samples was extracted from 100 μL of each sample, and then, 5 μL of the extracted nucleic acid was used for quantification using a commercial one-step RT-PCR kit (Genmagbio, Beijing, China). All real-time RT-PCR tests were performed using the CFX96TM Real-Time System and a C1000TM thermal cycler device (Bio-Rad Inc., Hercules, CA, USA). The RNA value was calculated in copies/mL by comparison with a standard curve of serial 10-fold dilutions of high titer plasmids of known potency in viral copy numbers. The sensitivity of real-time RT-PCR was ~8 copies per test and 800 copies/mL.

### Serological testing

HEV antigens were detected in the samples using commercial kits from Wantai, Beijing, China, which were optimized as previously reported^26^. The antibodies mAb 12F12^26^ and mAb no.4^28^ were used as the capture and detection antibodies, respectively. These commercial antigen detection kits can only be used for research. Anti-HEV antibodies (anti-HEV IgG/IgM) were detected using commercial ELISA kits (Wantai, Beijing, China) according to the manufacturer’s instructions. These commercial kits have been used in many studies, and anti-HEV IgG has been shown to be the most sensitive test available for anti-HEV IgG detection^2,29,30^. The levels of anti-HEV IgG were measured using a World Health Organization (WHO) reference serum^31^. The detection limit of the assay was 0.077 WHO units per milliliter (WU/mL)^31^. If the samples were negative for anti-HEV IgG detection, then the levels of anti-HEV IgG in these samples were set as 0.039 WU/mL. A 50-μL volume of each sample was used for antigen detection, and a volume of 10 μL was used for antibody detection.

The levels of immunoglobulin A (IgA), immunoglobulin M (IgM), and immunoglobulin G (IgG) in plasma samples of donors with persistent HEV viremia and/or antigenemia for ≥ 70 days after donation were quantified using commercial kits according to the manufacturer’s instructions (Bethyl, Texas, USA). Normal levels for IgA, IgM, and IgG were 0.8–4 g/L, 0.4–2.5 g/L, and 7–16 g/L, respectively^32,33^. HIV, HTLV, HAV, HBV, HCV, HDV, CMV, CA16, TB, and EV71 in plasma samples of donors with persistent HEV viremia and/or antigenemia for ≥ 70 days after donation were also measured in the lab using commercial kits according to the manufacturer’s instructions (Wantai, Beijing, China).

### HEV genotyping and sequence analysis

HEV RNA was amplified using different methods and primer pairs that targeted ORF1 (nt 119–364 and 4325-4603) or ORF2 (nt 5978-6389, 6028-6331, and 6351-6500, according to GenBank accession no. AJ272108) reported in previous studies^15,16,34–37^. After DNA sequencing, the sequences were analyzed against the available sequences in the GenBank database using Mega 6 software. The phylogenetic tree was analyzed using the neighbor-joining model.

### Statistical analysis

The statistical analysis was performed using SPSS 17 (SPSS Inc., Chicago, IL, USA). Differences between blood donors and HE patients were analyzed using the Mann–Whitney *U* test. Differences with a *p* value of < 0.05 were considered to be statistically significant.

## Electronic supplementary material

Supplemental_Materials

## References

[1] Dalton HR, Kamar N, Izopet J (2016). Hepatitis E in developed countries: current status and future perspectives. Future Microbiol..

[2] Vollmer, T., Diekmann, J., Eberhardt, M., Knabbe, C. & Dreier, J. Hepatitis E in blood donors: investigation of the natural course of asymptomatic infection, Germany, 2011. *Euro Surveill*. **21**, 10.2807/1560-7917.ES.2016.21.35.30332 (2016). 10.2807/1560-7917.ES.2016.21.35.30332 PMC5015460 27608433

[3] Skidmore SJ, Yarbough PO, Gabor KA, Reyes GR (1992). Hepatitis E virus: the cause of a waterbourne hepatitis outbreak. J. Med. Virol..

[4] Arankalle VA, Chobe LP (2000). Retrospective analysis of blood transfusion recipients: evidence for post-transfusion hepatitis E. Vox Sang..

[5] Haim-Boukobza S (2012). Transfusion-transmitted hepatitis E in a misleading context of autoimmunity and drug-induced toxicity. J. Hepatol..

[6] Boxall E (2006). Transfusion-transmitted hepatitis E in a ‘nonhyperendemic’ country. Transfus. Med..

[7] Matsubayashi K (2008). A case of transfusion-transmitted hepatitis E caused by blood from a donor infected with hepatitis E virus via zoonotic food-borne route. Transfusion.

[8] Hogema BM (2016). Incidence and duration of hepatitis E virus infection in Dutch blood donors. Transfusion.

[9] Tedder RS (2016). Virology, serology, and demography of hepatitis E viremic blood donors in South East England. Transfusion.

[10] Ren F (2014). Hepatitis E virus seroprevalence and molecular study among blood donors in China. Transfusion.

[11] Hewitt, P. E. et al. Hepatitis E virus in blood components: a prevalence and transmission study in southeast England. *Lancet*10.1016/s0140-6736(14)61034-5 (2014). 10.1016/S0140-6736(14)61034-5 25078306

[12] Satake M (2017). Unique clinical courses of transfusion-transmitted hepatitis E in patients with immunosuppression. Transfusion.

[13] Idolo A (2013). Identification of HEV in symptom-free migrants and environmental samples in Italy. J. Viral Hepat..

[14] Guo QS (2010). Prevalence of hepatitis E virus in Chinese blood donors. J. Clin. Microbiol..

[15] Li RC (2006). Seroprevalence of hepatitis E virus infection, rural southern People’s Republic of China. Emerg. Infect. Dis..

[16] Huang SJ (2010). Profile of acute infectious markers in sporadic hepatitis E. PLoS ONE.

[17] Baylis S, Koc Ouml, Nick S, Blümel J (2012). Widespread distribution of hepatitis E virus in plasma fractionation pools. Vox Sang..

[18] Lucarelli, C. et al. High prevalence of anti-hepatitis E virus antibodies among blood donors in central Italy, February to March 2014. *Euro Surveill*. **21**, 10.2807/1560-7917.ES.2016.21.30.30299 (2016). 10.2807/1560-7917.ES.2016.21.30.30299 27494608

[19] Kamar N (2012). Hepatitis E. Lancet.

[20] Myint KSA (2006). Hepatitis E antibody kinetics in Nepalese patients. Trans. R. Soc. Trop. Med. Hyg..

[21] Gupta E, Pandey P, Pandey S, Sharma MK, Sarin SK (2013). Role of hepatitis E virus antigen in confirming active viral replication in patients with acute viral hepatitis E infection. J. Clin. Virol..

[22] Kamar N (2011). Factors associated with chronic hepatitis in patients with hepatitis e virus infection who have received solid organ transplants. Gastroenterology.

[23] Geng Y (2016). Detection and assessment of infectivity of hepatitis E virus in urine. J. Hepatol..

[24] Westholter, D. et al. HEV positive blood donations represent a relevant infection risk for immunosuppressed recipients. *J. Hepatol*. 10.1016/j.jhep.2018.02.031 (2018). 10.1016/j.jhep.2018.02.031 29551705

[25] Jothikumar N, Cromeans TL, Robertson BH, Meng XJ, Hill VR (2006). A broadly reactive one-step real-time RT-PCR assay for rapid and sensitive detection of hepatitis E virus. J. Virol. Methods.

[26] Wen GP (2015). A valuable antigen detection method for diagnosis of acute hepatitis E. J. Clin. Microbiol..

[27] Zhao M (2015). A comprehensive study of neutralizing antigenic sites on the hepatitis e virus (hev) capsid by constructing, clustering, and characterizing a tool box. J. Biol. Chem..

[28] Zhang F (2006). Detection of HEV antigen as a novel marker for the diagnosis of hepatitis E. J. Med. Virol..

[29] Bendall R, Ellis V, Ijaz S, Ali R, Dalton H (2010). A comparison of two commercially available anti-HEV IgG kits and a re-evaluation of anti-HEV IgG seroprevalence data in developed countries. J. Med. Virol..

[30] Wenzel JJ, Preiss J, Schemmerer M, Huber B, Jilg W (2013). Test performance characteristics of Anti-HEV IgG assays strongly influence hepatitis E seroprevalence estimates. J. Infect. Dis..

[31] Zhu FC (2010). Efficacy and safety of a recombinant hepatitis E vaccine in healthy adults: a large-scale, randomised, double-blind placebo-controlled, phase 3 trial. Lancet.

[32] Chapel H, Cunningham-Rundles C (2009). Update in understanding common variable immunodeficiency disorders (CVIDs) and the management of patients with these conditions. Br. J. Haematol..

[33] Ameratunga R, Woon ST, Gillis D, Koopmans W, Steele R (2013). New diagnostic criteria for common variable immune deficiency (CVID), which may assist with decisions to treat with intravenous or subcutaneous immunoglobulin. Clin. Exp. Immunol..

[34] Ijaz S (2005). Non–travel-associated hepatitis E in England and Wales: demographic, clinical, and molecular epidemiological characteristics. J. Infect. Dis..

[35] Vollmer T (2012). Novel approach for detection of hepatitis E virus infection in German blood donors. J. Clin. Microbiol..

[36] Johne R (2010). Detection of a novel hepatitis E-like virus in faeces of wild rats using a nested broad-spectrum RT-PCR. J. Gen. Virol..

[37] Mizuo H (2002). Polyphyletic strains of hepatitis E virus are responsible for sporadic cases of acute hepatitis in Japan. J. Clin. Microbiol..

