# Supplementary material for: Long-term HEV carriers without antibody seroconversion among eligible immunocompetent blood donors
Source: Emerg Microbes Infect. 2018 Jul 5;7:125. doi: 10.1038/s41426-018-0125-y (PMC6033859; doi:10.1038/s41426-018-0125-y)
Supplement: Supplementary file 1 — Supplemental_Materials [file 41426_2018_125_MOESM1_ESM.pdf]

The concordance between the HEV RNA, HEV antigen, and anti-HEV IgM values was calculated using the Kappa test.

Table S1. Analyses of consistency between assays using the kappa index

		HEV Ag			Kappa (95% CI)	Anti-HEV IgM			Kappa (95% CI)
		Positive	Negative	Total		Positive	Negative	Total	
HEV RNA	Positive	11	4	15	0.73 (0.55-0.91) *	0	15	15	0.00 (-0.01-0.00)
	Negative	4	5326	5330		38	5292	5330	
	Total	15	5330	5345		38	5307	5345	
HEV IgM	Positive	0	38	38	0.00 (-0.01-0.00)				
	Negative	15	5292	5307					
	Total	15	5330	5345					

* $p<0.01$

Table S2. Characteristics of the donors with negative conversion of both HEV antigen and RNA

c	Age[a]	Sex[b]	Career[c]	Sample	Days post-donation	RNA (copies/mL)[d]	HEV Ag (S/CO)[e]	Anti-HEV IgM (S/CO)[e]	Anti-HEV IgG (WU/mL)[f]
11	46	M	Office clerk	11-1	1	2.78E+04	2.508	0.165	0.039
				11-2	21	neg	1.967	0.031	0.039
				11-3	202	neg	0.467	0.004	0.039
12	33	M	Office clerk	12-1	1	2.64E+04	1.042	0.069	0.326
				12-2	85	neg	0.617	0.188	0.210
13	23	F	Student	13-1	1	9.27E+02	1.900	0.027	0.039
				13-2	70	neg	0.608	0.019	0.039
14	24	M	Others	14-1	1	1.93E+05	1.467	0.062	0.039
				14-2	217	neg	0.375	0.054	0.039
15	23	F	Others	15-1	1	6.80E+04	1.075	0.031	0.039
				15-2	72	neg	0.117	0.121	0.039
16	36	M	Office clerk	16-1	1	3.26E+04	5.033	0.038	0.039
				16-2	95	neg	0.067	0.015	0.039
17	23	M	Student	17-1	1	2.77E+05	1.042	0.038	0.039
				17-2	142	neg	0.683	0.027	0.039

[a] Age at time of donation; [b] Sex: F: female; M: male.

[c] Potential careers included farmer, worker, office clerk, civil servant, student, teacher, doctor, other health worker, business staff, and other, with unemployed, self-employed, and housewife included in the “others” category.

[d] "neg" indicates negative for HEV RNA detection.

[e] S/CO: signal to cut-off ratios. Values of S/CO ≥1 were identified as positive.

[f] WU/mL: WHO units/mL. The levels of anti-HEV IgG in samples that were negative for anti-HEV IgG detection were set as 0.039 WU/mL.

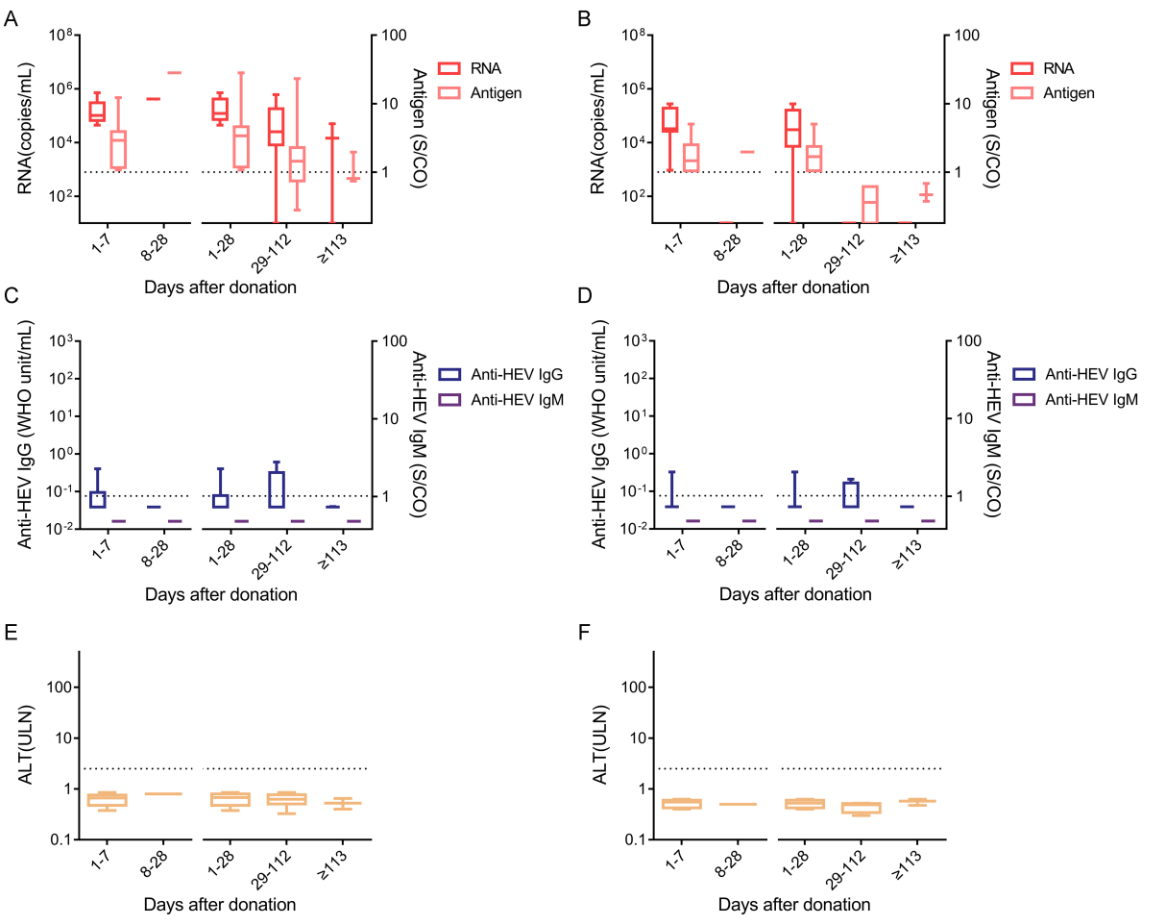

Figure S1. Dynamics of the HEV pathogen (RNA and antigen) (A and B), anti-HEV antibodies (IgM and IgG) (C, D), and ALT levels (E and F) among 10 donors with long-term HEV viremia and/or antigenemia (left panel) and 7 donors who cleared the virus (right panel) during the follow-up period. Samples were collected from 10 donors who presented with long-term HEV viremia and/or antigenemia from 1 to 7 days (n = 10), 8 to 28 days (n = 1), 1 to 28 days (n = 11), 29 to 112 days (n = 10), and ≥113 days (n = 3) after donation. Samples were collected from 7 donors who cleared the virus from 1 to 7 days (n = 7), 8 to 28 days (n = 1), 1 to 28 days (n = 8), 29 to 112 days (n = 4), and ≥113 days (n = 3) after onset of symptoms. Dotted lines represent the cut-off levels for HEV antigen

and anti-HEV IgM/IgG values. The levels of HEV-related markers are presented as ranges (whiskers), interquartile ranges (boxes), and medians (lines within the boxes).

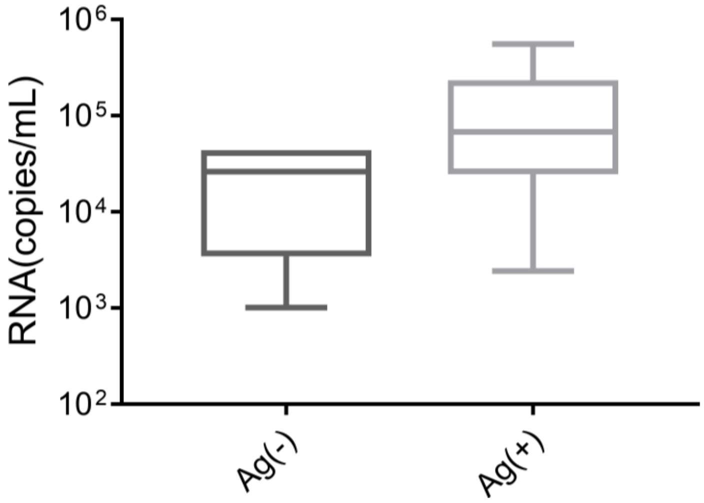

Figure S2. HEV RNA levels in donors with single HEV RNA positive (n=4) and both HEV RNA and antigen positive (n=11).
